# To be or Not to be Threatening, but What was the Question? Biased Face Evaluation in Social Anxiety and Depression Depends on How You Frame the Query

**DOI:** 10.3389/fpsyg.2013.00205

**Published:** 2013-04-30

**Authors:** Wolf-Gero Lange, Mike Rinck, Eni S. Becker

**Affiliations:** ^1^Department of Clinical Psychology, Behavioural Science Institute, NijCare, Radboud University NijmegenNijmegen, Netherlands

**Keywords:** social anxiety disorder, social phobia, depression, explicit evaluation, facial expression, methodology

## Abstract

Scientific evidence is equivocal on whether Social Anxiety Disorder (SAD) is characterized by a biased negative evaluation of facial expressions, even though it is assumed that such a bias plays a crucial role in the maintenance of the disorder. The way of framing the evaluation question may play an important role in the inconsistencies of earlier results. To investigate this issue, an unselected sample of 95 participants (11 males) with varying degrees of social anxiety and depressive symptoms rated facial crowds with different ratios of neutral-disgust, neutral-sad, neutral-happy, and neutral-surprised expressions in terms of friendliness, approval, difficulty to make contact, and threat. It appeared that the impact of social anxiety on ratings was highly dependent on the type of question that was asked, but not on the type of emotion that was shown: a high degree of social anxiety was related to a more positive evaluation of crowds when friendliness was assessed. When asking about the difficulty to make contact, social anxiety was related to more difficulty. When the threat evoked by a crowd had to be evaluated, higher degrees of social anxiety were tendentiously correlated with higher threat ratings. Degree of depression, on the other hand, was negatively correlated only to approval ratings. In addition, with an increasing degree of depression, the negative impact that any additional emotional face had on approval ratings increased as well. The theoretical and methodological implications of the results are discussed.

## Introduction

Social anxiety disorder (SAD) is a common, debilitating condition that is characterized by an excessive fear of scrutiny or negative evaluation by others (American Psychiatric Association, 2000). Cognitive models of SAD (Clark and Wells, 1995; Rapee and Heimberg, 1997) suggest that, especially the interpretation/evaluation of (ambiguous) social cues as negative or threatening, may play a predominant role in the maintenance and maybe even in the etiology of SAD (see also, Clark and Wells, 1995). Results from experimental research indicates that high degrees of social anxiety are indeed related to more negative interpretations of (written) ambiguous/neutral but also of positive and negative social situations or of comments by others in “video encounters” (Stopa and Clark, 2000; Voncken et al., 2003; Amir et al., 2005; Huppert et al., 2007). Facial expressions are believed to have evolved as means for communicating under more, anger, and fear, but also appraisal and disapproval in human social interaction (Darwin, 1872; Fridlund, 1994). As facial expressions can be quite ambiguous at times, it is assumed that they may form the most prominent social cues to undergo misinterpretation in SAD. From his literature review about face processing in social anxiety, Staugaard (2010) concluded that high socially anxious individuals (SAs) show an attentional bias to negative facial expressions: when presented with a variety of (task irrelevant) faces SAs seem to automatically focus their attention on the negative ones, while non-anxious controls (NACs) do either show no particular bias or tend to quickly focus on the positive expressions. It is assumed that cognitive biases such as “attentional biases” are fueled by an overactive “threat evaluation system” that generally tags fear-relevant cues in the environment as mostly threatening rather than non-threatening (Mogg and Bradley, 1998). Keeping this mechanism in mind, it is most surprising, that such a threat evaluation influences automatic attentional processes and behavioral avoidance impulses with respect to faces (Heuer et al., 2007; Lange et al., 2008; Roelofs et al., 2010), but not explicit evaluations of the very same stimuli (Staugaard, 2010).

In fact, findings about biased processing of facial expressions are the least coherent when explicit evaluations are assessed. For example, Dimberg and Christmanson (1991), found that high SAs rated angry faces as more negative than NACs, but did not differ when evaluating the pictures with regard to friendliness, hostility, or directedness. Using the same faces, Dimberg (1997) found different results: SAs evaluated happy faces as less positive and more hostile than NACs, but he did not find any rating differences on angry faces. Straube et al. (2004) found no difference in valence evaluations between SAD patients and NACs, but SAs reported more arousal in response to angry faces. A year later, Straube et al. (2005) reported no differences with regard to arousal, but found that SAD patients rated happy faces as more positive. Winton et al. (1995) reported a tendency of SAs to generally rate facial expressions as more negative, and Campbell et al. (2009) found a tendency of SAs to rate smiling faces as less approachable. Stevens et al. (2008) reported that SAs rated neutral and happy faces as less friendly than NACs. Neutral faces were also perceived as more rejecting. Gilboa-Schechtman et al. (2005) used grouped faces (crowds) to enhance ambiguity and thus susceptibility for biased interpretations. They showed that patients with SAD had a tendency to evaluate moderately disapproving crowds more negatively than NACs did. Individuals with SAD and comorbid depression evaluated extremely disapproving crowds more negatively.

Heuer et al. (2007), on the other hand, found no differences between SAs and NACs when rating smiling, angry, and neutral facial expression in terms of “pleasantness.” Douilliez and Philippot (2003) found no differences between SAs and NACs on the dimension “threatening,” when evaluating angry, joyful, and neutral faces. Our own previous work (Lange et al., 2008, 2011), did not substantiate any relation between degree of social anxiety and ratings of friendliness of emotional crowds, either. Although friendliness ratings decreased with increasing number of angry faces in a crowd, SAs and NACs did not differ in their evaluation. In the same line, Douilliez and Philippot (2003) found no differences between SAs and NACs on the dimension “threatening,” when it came to the evaluation of angry, joyful, and neutral faces. In a more systematic approach, Philippot and Douilliez (2005) instructed SAs and NACs to rate single emotional facial expressions portraying different degrees of joy, anger, fear, sadness, and disgust in terms of happiness, sadness, fear, anger, disgust, surprise, and shame. However, the authors found no group differences with regard to accuracy of categorization and intensity.

Yet, some conclusive results come from a small number of studies that explored evaluations after imagining an interaction with the depicted character and estimating the personal (emotional) *costs* that such an interaction may bring. Schofield et al. (2007), for example, asked SAs and NACs to rate what it would be like to interact with the depicted individual (i.e., “very good for me,” “very bad for me”). Results suggested that SAs, in comparison to NACs, found a hypothetical social interaction with someone expressing disgust (but not happy) more emotionally costly, regardless of the intensity of the emotion. In the same line, Douilliez et al. (2012) found that the more socially anxious the participants were, the higher they evaluated the emotional costs of interacting with single faces, independent of the displayed emotion (anger/neutral). When confronted with facial crowds, SAs showed a negative bias when estimating emotional costs of a hypothetical interaction, and when judging the level of disapproval, independent of emotion-neutral ratio.

In sum, no firm conclusions can be drawn about explicit face evaluation biases in SAs. It appears as if primarily methodological differences, rather than theoretical underpinnings, may account for most of the contradictions in these findings. In all studies reviewed above, different facial expressions or their combinations were used [e.g., happy-angry and angry-neutral crowds by Lange et al. (2008, 2011), happy, neutral, and angry expression combinations by Gilboa-Schechtman et al. (2005)] and, what is more, different evaluative dimensions were explored. For instance, Lange et al. (2008) asked the participants to evaluate the friendliness of facial crowds, while Douilliez et al. (2012) asked for ratings of disapproval and emotional cost. The latter, it appears, may, after all lead to the most conclusive results: investigating the elevated emotional cost ratings of SAs when confronted with faces. In addition, it seems that displays of single emotional expressions were less successful in detecting any other evaluation bias in SAs (except, Schofield et al., 2007; Douilliez et al., 2012).

Therefore, the objective of the present study was to investigate if SAs are negatively biased in their evaluation of (emotional) facial crowds, while taking the limitations of previous research into account. First, as groups of different faces are considered more susceptible to misinterpretation than single faces are (Gilboa-Schechtman et al., 2005), and as Douilliez et al. (2012) argued that grouped faces will evoke socially anxious concerns more distinctly, crowds were used for the present study. Second, in order to directly compare the impact of asking different evaluation questions, and to differentiate between social “costly” from non-costly questions, participants were asked to evaluate the presented crowds with respect to: “friendliness,” “difficulty to make contact (emotional cost),” “ threat when giving a presentation,” and “approval.” Third, while angry and neutral faces are repeatedly investigated stimulus emotions when social anxiety is concerned (Staugaard, 2010), the present study is the first one that also explored evaluations of less frequently studied emotions in facial crowds (i.e., disgust-neutral, happy-neutral, sad-neutral, and surprise-neutral). Fourth, the emotional intensity of a crowd was manipulated by presenting different ratios of emotion-neutral combinations. Due to the high comorbidity of social anxiety and depression, the latter was also assessed and controlled for.

The first hypothesis served as manipulation check: (1) ratings will, irrespective of question type, generally be most negative in neutral-disgust crowds. Neutral-sad crowds will be seen as more positive than neutral-disgust crowds, but more negative than neutral-surprise crowds. Neutral-smiling crowds will be seen as most positive. (2) In the same line, ratings are expected to become more negative with increasing intensity (ratio emotional/neutral faces) of disgust and sad crowds, but become more positive with increasing ratios of surprise or happy faces in neutral crowds. With regard to social anxiety, it is expected that (3) in line with Douilliez et al. (2012), cost ratings will increase with higher degrees of social anxiety, while approval ratings will decrease, independent of the presented crowd type and crowd ratio. No negative biases are expected on “threat” and “friendliness” ratings. (4) The biased rating of emotional cost and approval with higher degrees of social anxiety will be most pronounced in neutral-disgust, and neutral-smiling crowds, respectively. (5) Based on the work of Gilboa-Schechtman et al. (2005), it is expected that especially moderately negative crowds (here: ratio of three neutral and six emotional faces) evoke rating differences between high and low SAs.

## Materials and Methods

### Participants

After excluding four participants due to missing questionnaire data and one due to technical problems, 95 students from Radboud University Nijmegen participated in the study, 84 women (*M*_age_ = 21.45, SD = 2.77) and 11 men (*M*_age_ = 21.82, SD = 3.4). The whole sample consisted of 65% individuals from Dutch origin, 28% Germans and the rest from other countries. With 51% psychology was the predominant field of studies, followed by pedagogy (13%) and language and culture studies (5%). The remaining 31% was distributed about evenly across 21 different fields of studies. The experiment took about 30 min, and, after completion, participants received 5 Euro or course credit.

### Questionnaires

Before the computer task, participants completed a general screening instrument to assess sociodemographic information (e.g., age, gender, native language, education level). After the computer task, level of social anxiety was assessed with the Liebowitz Social Anxiety Scale (LSAS; Liebowitz, 1987; Oakman et al., 2003). Participants continued by completing the trait version of the State/Trait Anxiety Inventory (STAI; Spielberger et al., 1983), a 20-item inventory that assesses participants’ general anxiety predisposition; and the Social Interaction Anxiety Scale (SIAS; Mattick and Clarke, 1998), that measures anxiety related to initiating and maintaining social interactions. Further, depressive symptoms were assessed via the Center for Epidemiological Studies-Depression Scale (CES-D; Ratloff, 1977). Participants also completed the Fear of Negative Evaluation Scale (FNE; Leary, 1983; Duke et al., 2006), a 14-item scale that assesses the tendency to worry about others peoples’ evaluation. All questionnaires were digitalized beforehand to allow completion on the computer.

### Apparatus

The Rating task and questionnaires were programed with “Inquisit” (Millisecond Software, 2002; version 3.0.6.0), and conducted on a 451 MHz Intel Pentium III computer with 256 MB of RAM, running Windows XP Professional. The connected Monitor was a “Vision Master Pro 410” from Liama Electric Cooperation.

### Stimuli

To construct the different emotional crowds, color photos of actors portraying happy, disgusted, sad, neutral, and surprised expressions were taken from the Radboud Face Database (Langner et al., 2010) and from the Karolinska Directed Emotional Face database (KDEF; Lundqvist et al., 1998). Based on the validation data of both databases, only actors were selected that scored equally high (>80%) on the recognizability of each of the depicted emotions. This procedure resulted in a selection of 12 male actors (three from KDEF). The individual pictures were equalized with regard to background- and shirt-color of the actors, and were arranged in a 4 × 3 matrix to construct a crowd. Four crowd types were created: happy-neutral, sad-neutral, surprise-neutral, and disgust-neutral. In order to manipulate the level of threat, five types of ratios (emotion/neutral) were used to change the intensity of emotion in each crowd type: 12:0, 9:3, 6:6, 3:9, 0:12. A ratio of, e.g., 12:0 represents a crowd with 12 emotional faces and 0 neutral faces. Each crowd appeared in 72 mm × 99 mm on the screen.

### Procedure

Upon arrival at the Behavioral Science Institute (BSI) lab, participants were greeted and accompanied to one of the cubicles. They were seated approximately 50 cm away from a computer screen with a standard computer keyboard and a computer mouse in front of them. The participants were informed about the general nature of the test session, and were asked to give informed consent. After that, participants were asked to fill in the sociodemographic information on the computer. Then they were instructed to evaluate every visible crowd on the shown dimension as fast as possible, while all instructions would also appear on the computer monitor during the task. The experiment began when the participant pressed the space bar on the computer keyboard. At that moment, the experimenter left the laboratory to avoid distracting the participant. The participants were instructed to look at the center of the screen where a fixation cross, lasting for 500 ms, was followed by a facial crowd. Along with the crowd, one of the four possible questions was displayed under the crowd. The experiment consisted of four blocks. In each block, all variations of the crowds were paired with only one of the questions. It was assumed that evaluating the “friendliness” of a crowd would demand the least explanation, while evaluation of the other domains always asked for some imagination of a context. Therefore, the “friendliness” block was the first one for all participants. The other blocks were randomized. The questions per block were: “How friendly do you find this group?,” “How approving do you find this group?,” “How difficult would it be to make contact with the group?,” and “How threatening would it be to give a presentation in front of this group?” Each block displayed the four crowd types in five different ratios at random, while the position of the individuals varied in each crowd. Participants had to respond to each question by using the computer mouse to click on one value of the Likert-scale that was displayed at the bottom of the screen. The scale was ranging from 1 (not at all) to 10 (very much). After rating the displayed crowd, the next crowd appeared on the screen. Except for the all-neutral crowds, all other ratios were repeated four times per crowd type, resulting in 64 emotional trials per block. As the all-neutral crowds served as 0:12 ratio for *all* crowd types, they were only repeated four times. This summed up to 68 crowds per block and 272 crowds for the whole experiment. After completing the crowd-rating task, participants could take a short break. Then the participants were asked to complete the remaining questionnaires on the computer. At the end of the experiment, participants were debriefed, compensated for their effort, and thanked for their participation.

### Design

A 4 (Question Type: friendliness, difficulty to approach, threatening, approving) × 4 (Crowd Type: happy-neutral, sad-neutral, disgust-neutral, surprise-neutral) × 5 (Expression Ratio: 12:0, 9:3, 6:6, 3:9, 0:12) factorial design, with social anxiety (LSAS total) and depression (CES-D total) as covariates was used for the analysis of the subjective ratings. Question, crowd type and ratio were within-subjects factors. Whenever the basic assumption of univariate testing (i.e., sphericity) was violated in any of the analyses, appropriate, more conservative tests with corrections of degrees of freedom were used (i.e., Huynh–Feldt). Additionally, because of the high number of levels of the “ratio” factor, we analyzed the contrasts/gradients of the resulting regression lines rather than the mean ratings per individual ratio. Whenever necessary, appropriate follow-up analyses such as correlational analyses or *t*-tests were conducted.

Note that high scores on “friendliness” and “approval” ratings originally meant more positive ratings and high scores on “difficulty to approach” and “threat” actually meant more negative ratings. In order to make the scales more comparable, all ratings in response to the “difficulty to approach” and the “threat” questions were recoded. Now all high scores represent a positive evaluation, more friendliness, more approval, less difficulty to approach, and less threat, while low scores refer to unfriendliness, less approval, more difficulty to approach, and more threat. An alpha level of 0.05 was used for all statistical tests unless repeated comparisons asked for Bonferroni-correction in the follow-up analyses.

## Results

### Participant characteristics

As can be seen in Table 1, participants scored in a medium range on all questionnaires and no gender differences occurred, all *t*’s < 1.34, all *p*’s > 0.18.

**Table 1:** **Means (*M*) and standard deviations (SD) of questionnaire scores for female and male participants: total scores of Liebowitz Social Anxiety Scale (LSAS), the trait version of the Spielberger State/Trait Anxiety Inventory (STAI-Trait), Social Interaction Anxiety Scale (SIAS), Center of Emotional Studies-Depression Scale (CES-D), and Fear of Negative Evaluation (FNE)**.

Variables	Gender			
	Female (*n* = 84)		Male (*n* = 11)	
	*M*	SD	*M*	SD
age	21.5	2.8	21.8	3.4
LSAS	36.7	19.1	29.8	11.4
STAI-trait	38.8	9.8	35.5	6.3
SIAS	22.5	13.1	18.8	10.3
CES-D	13.8	8.9	10.1	4.6
FNE	24.7	6.9	23.6	7.6

### Crowd ratings – overall analysis

In the first analysis there was a main effect of question type, *F*(3, 237.7) = 23.25, *p* < 0.001, ∂η^2^ = 0.20. As expected, crowd type, *F*(3, 209.79) = 123.16, *p* < 0.001, ∂η^2^ = 0.57, and expression ratio, *F*(4, 118.73) = 6.88, *p* < 0.001, ∂η^2^ = 0.07, also yielded significant main effects. Neither of the covariates (LSAS nor CES-D) reached a significant main effect, both *F*’s < 1.85, both *p*’s > 0.18.

Follow-up analyses for the main effects revealed that the averaged ratings across question types differed between all questions (1 friendliness, 2 difficulty to make contact, 3 threat when presenting, 4 approval), except for question 1 and 4 (Table 2). While question 4 delivered the most negative evaluations (*M* = 4.5, SE = 0.07), followed by question 1 (*M* = 4.6, SE = 0.09), more positive evaluations were given after question 2 (*M* = 5.4, SE = 0.9), and question 3 (*M* = 5.8, SE = 0.11). In addition, all crowd types differed significantly from one another and in the expected way: disgusted crowds (*M* = 3.6, SE = 0.08) were evaluated most negatively, followed by sad crowds (*M* = 4.6, SE = 0.8). Surprised faces (*M* = 5.0, SE = 0.08) were evaluated more positively and happy crowds (*M* = 7.1, SE = 0. 08) were evaluated as most positive (Table 3). Finally, the linear contrast (slope) for the main effect of expression ratio revealed that ratings gradually declined with an increasing number of emotional faces in the crowds, *F*(1, 92) = 7.1, *p* < 0.01, ∂η^2^ = 0.07.

**Table 2:** **Mean difference scores (*M*_Diff_), standard errors (SE), and *p*-values for each pairwise comparison of question type: question 1 (friendliness; FRIEND), question 2 (Difficulty to make contact; DIFFICULT), question 3 (Threat to give presentation; THREAT), and question 4 (approval of group; APPROVE)**.

Comparison (questions)		*M*_Diff_	SE	*p*
FRIEND vs.	DIFFICULT	−0.81	0.11	<0.001
	THREAT	−1.21	0.14	<0.001
	APPROVE	0.06	0.09	ns
DIFFICULT vs.	THREAT	−0.40	0.12	<0.01
	APPROVE	0.87	0.09	<0.001
THREAT vs.	APPROVE	1.27	0.12	<0.001

*The comparisons are Bonferroni-corrected*.

**Table 3:** **Mean difference scores (*M*_Diff_), standard errors (SE), and *p*-values for each pairwise comparison of crowd type**.

Comparison (crowd type)		*M*_Diff_	SE	*p*
Disgust-neutral	Happy-neutral	−3.50	0.12	<0.001
	Sad-neutral	−0.96	0.07	<0.001
	Surprise-neutral	−1.42	0.09	<0.001
Happy-neutral	Sad-neutral	2.53	0.10	<0.001
	Surprise-neutral	2.08	0.09	<0.001
Sad-neutral	Surprise-neutral	−0.45	0.07	<0.001

*The comparisons are Bonferroni-corrected*.

Further, significant interactions with question type indicated the important role of this factor: question type × crowd type, *F*(7.5, 689.86) = 3.49, *p* = 0.001, ∂η^2^ = 0.04, question type × expression ratio, *F*(5.38, 495.12) = 9.42, *p* < 0.001, ∂η^2^ = 0.09, and question type × crowd type × expression ratio, *F*(23.75, 2184.63) = 1.83, *p* < 0.01, ∂η^2^ = 0.02. The interaction of crowd type × expression ratio, *F*(4.69, 431.55) = 78.98, *p* < 0.001, ∂η^2^ = 0.46, was also significant but will not be explored in more detail as it is irrelevant for the research questions. As for the covariates, question type significantly interacted with degree of social anxiety, *F*(76.06, 237.70) = 5.49, *p* < 0.01, ∂η^2^ = 0.06, and question type × expression ratio interacted with degree of depression, *F*(5.38, 495.12) = 2.68, *p* = 0.02, ∂η^2^ = 0.03. All other interactions were not significant, all *F*’s < 1.9, all *p*’s > 0.09. For means and standard errors, see Table 4.

**Table 4:** **Mean scores, standard errors (in parentheses) per question (friendliness, FRIEND; Difficulty to make contact, DIFFICULT; Threat when giving a presentation; THREAT; approval by crowd, APPROVE), crowd type, and expression ratio (12:0 all-emotion: zero neutral to 0:12 zero emotion: all-neutral)**.

	Disgust-neutral					Happy-neutral				
	12:00	9:3	6:6	3:9	0:12	12:00	9:3	6:6	3:9	0:12
FRIEND	2.36 (0.12)	2.70 (0.12)	3.12 (0.11)	3.54 (0.11)	4.63 (0.15)	8.50 (0.13)	7.30 (0.12)	6.55 (0.11)	5.75 (0.12)	4.57 (0.14)
DIFFICULT	2.60 (0.16)	3.27 (0.15)	3.63 (0.14)	4.32 (0.13)	5.79 (0.17)	8.82 (0.15)	7.95 (0.13)	7.48 (0.12)	6.96 (0.13)	5.76 (0.17)
THREAT	2.83 (0.20)	3.31 (0.17)	3.88 (0.17)	4.68 (0.16)	6.45 (0.19)	8.73 (0.15)	8.16 (0.14)	7.88 (0.12)	7.45 (0.13)	6.44 (0.20)
APPROVE	1.89 (0.12)	2.26 (0.11)	2.76 (0.12)	3.38 (0.11)	4.99 (0.16)	8.60 (0.14)	7.46 (0.13)	6.78 (0.12)	6.23 (0.11)	4.91 (0.15)

	**Sad-neutral**					**Surprise-neutral**				
	**12:00**	**9:3**	**6:6**	**3:9**	**0:12**	**12:00**	**9:3**	**6:6**	**3:9**	**0:12**

FRIEND	3.69 (0.15)	3.79 (0.14)	4.04 (0.11)	4.25 (0.11)	4.54 (0.16)	4.69 (0.15)	4.52 (0.13)	4.51 (0.13)	4.37 (0.12)	4.51 (0.14)
DIFFICULT	4.38 (0.17)	4.66 (0.14)	4.84 (0.14)	5.27 (0.13)	5.60 (0.18)	5.13 (0.16)	5.26 (0.14)	5.25 (0.12)	5.56 (0.12)	5.66 (0.17)
THREAT	4.82 (0.19)	5.05 (0.16)	5.40 (0.15)	5.73 (0.15)	6.43 (0.19)	5.42 (0.20)	5.55 (0.18)	5.63 (0.16)	5.86 (0.14)	6.48 (0.21)
APPROVE	2.96 (0.12)	3.39 (0.12)	3.76 (0.10)	4.16 (0.11)	4.92 (0.13)	4.25 (0.16)	4.32 (0.13)	4.39 (0.12)	4.48 (0.10)	4.88 (0.16)

*Covariates appearing in the model are evaluated at the following values: LSAS = 35.86, CES = 13.35. Due to recoding, higher scores refer to less threat/difficulty, while low scores represent more threat/difficulty*.

To examine the interactions specifically related to the research questions, separate analyses were conducted per question type.

### Friendliness

As in the overall analysis, main effects of crowd type, *F*(2.45, 224.90) = 115.23, *p* < 0.001, ∂η^2^ = 0.56, and expression ratio, *F*(1.69, 155.48) = 3.75, *p* = 0.03, ∂η^2^ = 0.04, occurred. Both main effects were comparable to those found in the overall analysis. Social anxiety as measured with the LSAS, reached a significant main effect, *F*(1, 92) = 4.61, *p* = 0.03, ∂η^2^ = 0.05, while degree of depressive symptoms as measured with the CES-D did not, *F*(1, 92) = 2.09, *p* = 0.15. Subsequent correlational analyses revealed that degree of social anxiety had a tendency to correlate positively with friendliness ratings: the higher anxiety was the more positive the ratings seemed to become, irrespective of emotion or ratio, *r*(95) = 0.171, *p* = 0.09. The only interaction that became significant was the one between crowd type and expression ratio, *F*(7.02, 645.61) = 49.18, *p* < 0.001, ∂η^2^ = 0.35. All other *F*’s < 2.09, all *p*’s > 0.12.

### Difficulty to make contact

Again, main effects of crowd type, *F*(2.32, 212.78) = 65.48, *p* < 0.001, ∂η^2^ = 0.42, and expression ratio, *F*(1.58, 145.43) = 4.39, *p* < 0.01, ∂η^2^ = 0.05, occurred. Social anxiety reached a significant main effect, *F*(1, 92) = 4.19, *p* = 0.04, ∂η^2^ = 0.04, while degree of depressive symptoms as measured with the CES-D did not, *F*(1, 92) < 0.01, *p* = 0.96. Subsequent correlational analyses revealed that degree of social anxiety was not significantly correlated with difficulty ratings, *r*(95) = −0.041, *p* = 0.69. Again, the interaction between crowd type and expression ratio, *F*(6.66, 612.37) = 34.07, *p* < 0.001, ∂η^2^ = 0.27 was significant. All other *F*’s < 1.60, all *p*’s > 0.20.

### Threat when giving presentation

Here, main effects of crowd type, *F*(2.51, 230.71) = 43.71, *p* < 0.001, ∂η^2^ = 0.32, and expression ratio, *F*(1.44, 132.56) = 11.70, *p* < 0.001, ∂η^2^ = 0.11, occurred. Social anxiety reached a marginally significant main effect, *F*(1, 92) = 3.33, *p* = 0.07, ∂η^2^ = 0.04, while degree of depressive symptoms did not, *F*(1, 92) < 0.10, *p* = 0.76. Subsequent correlational analyses revealed that degree of social anxiety was negatively correlated with threat ratings: the higher anxiety was, the more threatening crowds were evaluated, irrespective of emotion or expression ratio, *r*(95) = −0.221, *p* = 0.03. Again, only the interaction between crowd type and expression ratio was significant, *F*(6.42, 590.86) = 19.12, *p* < 0.001, ∂η^2^ = 0.17. All other *F*’s < 1.05, all *p*’s > 0.39.

### Approval

As in the analyses above the effects of crowd type, *F*(2.43, 223.32) = 118.20, *p* < 0.001, ∂η^2^ = 0.56, and expression ratio, *F*(1.80, 165.32) = 10.32, *p* < 0.001, ∂η^2^ = 0.10, were significant. Social anxiety had no significant main effect, *F*(1, 92) = 0.11, *p* = 0.74, while degree of depressive symptoms had, *F*(1, 92) = 5.24, *p* = 0.02, ∂η^2^ = 0.05. Subsequent correlational analyses revealed that degree of depression was negatively correlated with approval ratings: the higher depression was, the less approving the crowds were evaluated, irrespective of emotion or expression ratio, *r*(95) = −0.24, *p* = 0.02.

The interaction between crowd type and expression ratio was significant, *F*(6.54, 601.47) = 38.72, *p* < 0.001, ∂η^2^ = 0.30 and the effects were comparable to those observed before. In addition, the interaction between expression ratio and depression was also significant, *F*(1.8, 165.32) = 8.35, *p* < 0.001, ∂η^2^ = 0.08. Here, subsequent analyses revealed that, with an increasing number of emotional expression in the crowds, the linear change of the approval ratings (slope) correlated significantly negatively with the depression scores, *r*(95) = −0.32, *p* < 0.01. This means that with an increasing degree of depression, the negative impact that any additional emotional face had on approval ratings, increased as well. All other *F*’s < 1.74, all *p*’s > 0.17.

## Discussion

The present study examined whether social anxiety is associated with biases in the explicit evaluation of a variety of facial crowds. Specifically, it was investigated in how far the type of evaluative dimension amplifies such a bias. The general results confirmed that the manipulation worked as expected: across all dimensions, the disgust-neutral crowds were evaluated as most negative. The sad-neutral crowds were evaluated more positively than that, but still more negatively than the surprised faces, which were in turn rated more negatively than the happy crowds. In addition, it was confirmed that, the ratings became gradually more negative when more negative expressions were added to the crowds and became more positive, when positive faces were added.

Most importantly, the hypothesis that the impact of social anxiety on explicit ratings of facial crowds depends on the rating dimension was confirmed by the results. Contrary to expectations, however, it was found that higher degrees of social anxiety were related to *higher* friendliness ratings of the crowds, irrespective of expression ratio or presented emotion. When asked for the difficulty to make contact, social anxiety did evoke a main effect, but no linear correlation with ratings was observed. When evaluating how threatening it would be to give a presentation in front of the particular crowd, ratings were as expected: participants’ social anxiety was correlated with higher threat evaluations. Evaluation of approval was by no means related to social anxiety scores. Instead, higher depression scores were correlated with lower approval ratings, irrespective of emotion and expression ratio. In addition, depression scores and the ratio of the presented emotions and neutral faces determined the evaluation of approval: higher depression scores led to steeper declines in the evaluations of approval when the ratios gradually changed, again irrespective of emotion.

### Friendliness ratings

Averaged across crowd type and ratio, increasing degrees of social anxiety were related to linearly increasing ratings of friendliness. In light of our own previous work, this is rather surprising. In two of our earlier studies (Lange et al., 2008, 2011) we did not find evidence for a relationship between social anxiety and these ratings whatsoever. To our knowledge, only single face studies had further evaluated friendliness, or possibly comparable dimensions such as pleasantness, or positive/negative valence with respect to social anxiety. Here, Amir et al. (2005), Campbell et al. (2009), Heuer et al. (2007), Merckelbach et al. (1989), Mühlberger et al. (2009), Schofield et al. (2007), Stevens et al. (2008), Straube et al. (2004), Vrana and Gross (2004) did not report any rating differences on the afore mentioned dimensions. Only Dimberg (1997) found more negative evaluations on smiling faces. On the other hand, Straube et al. (2005) reported that SAs saw smiling faces as more pleasant. In the light of current cognitive models (Clark and Wells, 1995; Rapee and Heimberg, 1997), it would have been expected that social anxiety may have a *negative* impact on the subjective ratings of friendliness, while evidence is obviously meager so far. In fact, there are more studies that do not provide evidence for a distorted friendliness-evaluation than studies that do. The two studies that did find significant differences, though in opposite directions, are characterized by particularly small sample sizes (Dimberg, 1997: 8 SAs vs. 8 NACs; Straube et al., 2005: 9 SAs vs. 9 NACs). The present results *do* provide evidence for a positive bias comparable to the one observed by Straube et al. (2005), but in our study, the effect size was rather small (η^2^ = 0.05), while the sample size was large (*N* = 95). It is in fact most likely that the significant findings are a statistical artifact rather than theoretically relevant results. Of course, it is possible that, as SAs are more sensitive to social demand effects, they may have considered crowds generally as more friendly, or at least, thought that it was expected of them to answer accordingly. By doing so, they may have over-exaggerated, not knowing the “normal” evaluation. In general, in these kinds of studies, evaluation of stimuli is part of a whole battery of tasks or serves as a manipulation check, rather than being the main focus of interest. In the present study, however, evaluation *was* the main interest of the study. This may have led to a thorough evaluation of the stimuli making the underlying cognitive distortions more explicit. The fact that the friendliness rating block was always presented first may have enhanced that effect.

### Difficulty to make contact

When evaluating the emotional costs that a possible interaction with the presented crowds would bring, social anxiety seemed to play a significant role. To a certain degree, this reflects and goes beyond the findings of Douilliez et al. (2012). They found that the more socially anxious the participants were, the more they evaluated a hypothetical social interaction with individuals in angry-neutral crowds as emotionally more costly, stable over different threat levels (i.e., ratios). The present results reflect this effect across all ratios as well, and in addition across all emotions involved. Seemingly, SAs find an interaction with a group of people more emotionally costly in general. In contrast to the previously mentioned friendliness ratings, more studies have reported a significant effect of social anxiety on emotional cost estimations (e.g., Vrana and Gross, 2004; Schofield et al., 2007; Douilliez et al., 2012). All of them confirmed that neither the type of displayed facial expression nor the intensity of the emotion or the ratio of this expression in the crowd, mattered in order to “evoke” the bias. Apparently, SAs consider *all* facial expressions as difficult to make contact with, at least when a hypothetical interaction with the people presented in the stimuli has to be imagined (Douilliez et al., 2012). This makes sense when considering SAs’ major fear of possible scrutiny by others. In lab research with static picture materials, this fear may only be evoked when the participants have to imagine an interaction with the presented people, and even more so when the initiative for the interaction lies with the participant (Lange et al., 2008). When asked to judge the difficulty to make contact, it is implied that the participant imagines him/herself to make/anticipate that contact and interrogate his/her own feelings about the difficulty of this move. If that is truly the case, it becomes even more relevant to have a close look at what evaluation question is used when explicit face evaluation biases are explored. Note, however, that although the general findings for emotional costs are in line with previous findings, the *post hoc* analyses could not substantiate a *significant* negative correlation between degree of social anxiety and the rated difficulty to make contact. Therefore, any conclusions have to be drawn very cautiously.

### Threat when giving a presentation

With increasing degrees of social anxiety, participants tendentiously overestimated the degree of threat in all crowd types, irrespective of expression ratio. Strictly speaking, this should be considered statistically not relevant as the related main effect was approaching but not reaching significance. Yet, in the light of the ongoing discussion about the formulation of the evaluation question, this is noteworthy nonetheless. The question was not merely “how threatening” do you find this crowd. This would be comparable to research with single face stimuli of Coles and Heimberg (2005), Dimberg and Christmanson (1991), Dimberg and Karlsson (1997), and Stein et al. (2002) who asked their participants about how critical, hostile, or harsh the face stimuli looked. Here, only Dimberg (1997) found a negative bias in SAs. The inclusion of an explicit *threat* rating of facial crowds in the present study followed a suggestion by Lange et al. (2008). They did not find an explicit evaluation bias in SAs’ friendliness ratings and noted that “friendliness” may not be social anxiety relevant enough. “Threat,” on the other hand, may be clearly anxiety related and therefore more capable to influence direct ratings, at least when evaluating a crowd of faces. In the present study, however, “threat when giving a presentation in front of these crowds” was assessed. As with the assessment of social costs, the addition of the “interaction” component “giving a presentation” may have triggered a negatively biased evaluation that a pure threat-question may not have had. This would in turn speak against the presumption of Lange et al. (2008) that explicit negatively biased evaluations of faces are not measured due to their cognitive inaccessibility, but that the accessibility dependents on the personal relevance for the one evaluating. Imagining personal interaction seems to increase the social relevance thereby triggering the context cues necessary to evoke biased cognitions.

### Approval ratings

In contrast to the hypothesis, social anxiety was not associated with lower ratings of approval by facial crowds. This is not in line with former work of Gilboa-Schechtman et al. (2005) who found that patients with generalized SAD evaluated moderately disapproving crowds (i.e., crowds containing four angry, two happy, and three neutral faces) as more disapproving than did NACs. Further, Douilliez et al. (2012) found that high degrees of social anxiety were associated with increased “disapproval”-ratings, and that threat intensity (ratio between neutral and angry faces) mediated this interaction even more. As the present study did not include angry faces, but focused on other emotions combined with neutral, it is possible that angry faces are specifically apt to trigger biased disapproval ratings, while other emotions are not. Angry expressions are an evolutionarily relevant and direct signal of dawning hostility (Fridlund, 1994) and therefore may elicit the strongest fear reactions in SAs, who generally view themselves as less dominant than others (Staugaard, 2010). Such a strong fear reaction could especially activate biases in information processing. In social interactions, however, the occurrence of angry faces, let alone an angry crowd, is rather unlikely. Since approval by others is one of the core elements of what social anxiety is about, a negative bias when judging approval only in angry faces is not plausible. Instead, it may be that, again, the framing of the question could be the determining factor: the studies by Gilboa-Schechtman et al. (2005) and Douilliez et al. (2012) both asked for “disapproval” while the present study asked for “approval.” It is possible that negative framing of the question, maybe in combination with “interaction” components, may readily trigger negative biases, while positive framing such as e.g., friendliness, pleasantness, acceptance, or approval may lie already beyond the “scale” that SAs would use with respect to evaluations of themselves by others. Negative formulations, such as how threatening, harsh, critical, rejecting, etc., a crowd is, may be part of the negative evaluation schemata that SAs readily handle and that scare them (Beck et al., 1985).

In light of the above, it is even more surprising that not social anxiety but degree of depression influenced the way in which participants evaluated the approval of crowds. It appeared that high depression scores were related to lower approval ratings, but also that the decline of ratings from one expression ratio to the next was steeper. Thus, degree of depression determined the impact that additional expressive faces in a crowd had on approval ratings. While this is an intriguing finding by itself that is truly worth discussing, it is, yet, beyond the scope of this article and will therefore be omitted.

### Limitations

The present results have to be seen in light of a number of limitations. First, although the order of the blocks for the evaluations of “difficulty to make contact,” “approval,” and “threat” were randomized for each participant, the first block always assessed the “friendliness” of the crowds. It is possible that the answers in this first block were more reliable as the participants may still have been motivated, while the motivation may have decreased in the consecutive blocks with 204 crowds to go. Despite the fact that we did find results in the remainder of the task, it would have been more elegant to randomize all four blocks to distribute diminishing motivation across all blocks.

Second, it cannot be ruled out that the observed evaluation differences are mediated by attentional biases. The literature suggests that SAs focus their attention primarily on negative faces, disregarding neutral ones (Staugaard, 2010). In that case, it would not be surprising if their evaluation turned out to be more negative. Douilliez et al. (2012) suggested to combine face evaluation with eye-tracking methods to control for attentional biases. Yet, Lange et al. (2011) did combine crowd evaluation with eye-tracking and did not find any association between the two.

Third, the fact that the stimuli included only male pictures, while participants were predominantly female, hampers the generalizability of the results considerably. Though we have reasons to believe that cross-gender interactions may trigger social anxiety more readily in a heterosexual sample, it would have been more elegant to have a more balanced gender distribution in the sample and have male as well as female crowd stimuli. Despite our attempts to equalize the pictures with respect to background, shirt-color, etc., it was not ideal to mix pictures of different databases. In the same line, it would have been more elegant to balance the mean individual expression intensity across crowd images.

Finally, it has to be noted that the correlational design used in this study is suboptimal. Although it allows for conclusions about the direction and strength of the observed associations, it does not allow for any conclusions about causality. Moreover, in the light of the rather small effect sizes, it may have been statistically more appropriate to use linear regression analyses or hierarchical linear models, though, with this high number of conditions/layers of factors, a much larger number of participants would have been necessary.

## Conclusion

In sum, it can be concluded that the degree of social anxiety is related to biased evaluation of facial crowds, irrespective of the depicted emotion, once the framing of the evaluation question is taken into account: high scores of social anxiety (a) correlated with high friendliness ratings, (b) seemed to be related to higher emotional cost ratings, and (c) were tendentiously correlated with higher threat ratings. From the results of the present study, it becomes clear that studying explicit face evaluation biases in social anxiety is a tedious enterprise while results with respect to attentional biases in social anxiety are fairly conclusive. It appears, that discussing explicit face evaluation biases and their contribution to the mechanisms underlying SAD is somewhat premature. First, a number of methodological problems have to be solved. The present research as well as that of Douilliez et al. (2012) seem to indicate that, when investigating explicit processing biases in social anxiety, using crowd stimuli may be the most promising path to follow. Yet, it has also become clear that probably the most crucial factor in this kind of research is the formulation of the evaluation question. Here, the distinction between self-relevant or not (e.g., “how threatening is the crowd to me” vs. “how threatening is the crowd”), positive or negative formulation (e.g., “approval” vs. “disapproval”), or imagining oneself in interaction with the crowd (e.g., “when giving a presentation in front of them”) or not, may make all the difference. Increasing the self-relevance (emotional cost) of the stimuli may even be *the* most crucial factor. As the present research has shown, these nuances may even exceed the influence that specific types of facial expressions in a crowd may have. More systematic research is needed to explore the methodological fine-tuning, to detect explicit face evaluation biases in SAD, and to detect their potential causal, maintaining and eventually therapeutic significance.

## Conflict of Interest Statement

The authors declare that the research was conducted in the absence of any commercial or financial relationships that could be construed as a potential conflict of interest.
